# Machiavellianism, level of personality functioning, and maladaptive personality traits: mediation analyses in a clinical sample

**DOI:** 10.3389/fpsyt.2026.1675044

**Published:** 2026-04-30

**Authors:** András Láng, Tibor Cece Kiss, Botond László Kiss, Diána Ágnes Láng

**Affiliations:** 1 Institute of Psychology, Faculty of Humanities and Social Sciences, University of Pécs, Pécs, Hungary; 2 Doctoral School of Psychology, University of Pécs, Pécs, Hungary; 3 Department of Psychosomatic and Psychotherapeutic Rehabilitation “Tündérhegy”, Semmelweis University, Rehabilitation Clinic, Budapest, Hungary

**Keywords:** level of personality functioning, machiavellian tactics, machiavellian views, machiavellianism, maladaptive personality traits, mediation analysis

## Abstract

**Introduction:**

Machiavellianism was repeatedly found to be associated with personality dysfunction. The Alternative Model for Personality Disorders offers a dimensional approach to personality disorders including level of personality functioning and maladaptive personality traits. The aim of this study was to test mediation models wherein level of personality functioning was suggested to mediate the relationship between maladaptive personality traits and Machiavellian views and tactics.

**Method:**

Using self-report measures, 341 mental health patients (Mage = 34.78 years; SDage = 10.99) of an inpatient psychotherapy ward participated in the study with various mental disorders. 200 patients identified as females, 94 as males, 1 as non-binary, 46 participants didn’t report their gender. Participation was anonymous and voluntary. All patients gave their informed consent.

**Results:**

Correlational analyses revealed that impairment in level of personality functioning and all maladaptive personality trait domains were positively associated with Machiavellian views and tactics (except for the nonsignificant association between Negative Affectivity and Machiavellian Tactics). According to results of mediational analyses, maladaptive personality traits of Detachment, Psychoticism, and Negative Affectivity were associated with Machiavellian views via indirect pathways through level of personality functioning, whereas Negative Affectivity and Antagonism were directly associated with Machiavellian tactics.

**Discussion:**

Correlational results were in line with existing literature. Correlational results and mediation analyses supported the distinct characteristics of Machiavellian views and tactics. The results of this study could be conclusive both for clinical practice and a better understanding of the concept of Machiavellianism.

## 1 Introduction

In its original conceptualization (1), Machiavellianism is a personality trait characterized by exploitative interpersonal tendencies of manipulation, cynical view of the world and human nature, and a utilitarian moral stance. A contemporary conceptualization (2–4) sees Machiavellianism as composed of two distinct but interrelated dimensions. Views represent the cognitive-emotional aspect, while Tactics represent the cognitive-behavioral aspect of Machiavellianism. Views capture an unflattering and pessimistic view of humanity, which is considered gullible, untrustworthy, selfish, and manipulative. At the same time, Tactics reflect an individual’s willingness to use any means, irrespective of morality, to achieve their goals. While Christie and Geis (1, p. 42.) argued that “there should be little or no relationship between Machiavellian orientations and measures of psychopathology”, recent body of research proved Machiavellianism to be related to maladaptive^1^ personality traits and personality dysfunction.

Current conceptualizations of personality disorders (PDs) advocate a dimensional approach – as opposed to categorical (for a recent overview see 5). Contrasting the categorical approach of DSM-5 (6) and ICD’s prior version (ICD-10; (7), ICD-11 (8) “focuses on global and shared features that apply to all PDs” (9, p.2.). The presence of a PD diagnosis is evaluated globally with regard to self and interpersonal functioning, cognitive, emotional, and behavioral characteristics, and distress and impairment of psychosocial functioning. Severity of personality disorder can range from mild to severe with an optional subdiagnostic code for Personality Difficulty to inform treatment and prevention. To express heterogeneity at different levels of PD severity, any number of trait domain specifiers can be applied to best capture the individual characteristics of the person assessed. Trait domain specifiers include five trait domains (Negative Affectivity, Detachment, Dissociality, Disinhibition, and Anankastia) that are continuous with the five-factor model of personality and a Borderline Pattern (9, 10).

Complementing and going beyond the categorical approach (11–13), the Alternative Model for Personality Disorders (AMPD; 14), was introduced in Section III of DSM-5 (6). AMPD suggested a dimensional approach to PDs, wherein criterion A of PDs was reconceptualized as the continuous dimension of level of personality functioning. Criterion B was replaced by different configurations of pathological personality traits. In AMPD, levels of impairment in personality functioning are assessed on a 4-point scale from little or no impairment to extreme impairment (6, 15, 16). Impairments can be divided into self-related (i.e., concerning identity and self-directedness) and interpersonal (i.e., concerning empathy and intimacy) problems (6). Prior research has already proven the relevance of both self-related functioning (17–23) and interpersonal functioning (24–30) for personality pathology. At the same time, personality functioning is conceptualized as a single dimension in AMPD, irrespective of the diverse empirical structures of personality functioning measures (31; for a summary see 32).

Instead of using a set of symptoms for Criterion B (i.e., specific personality disorders symptoms), AMPD utilizes personality trait configurations composed of traits from five domains: Negative Affectivity, Detachment, Antagonism, Disinhibition, and Psychoticism (33, 34). The above five domains are suggested as the maladaptive counterparts of universally accepted and evidence-based five-factor personality model dimensions (35). Accordingly, a strong positive relationship was found between Negative Affectivity and Neuroticism, while a negative relationship was found between Detachment and Extraversion, Antagonism and Agreeableness, and Disinhibition and Conscientiousness (36–38). The relationship between Psychoticism and Openness was found to be inconsistent (39), Psychoticism reflecting peculiarities in thinking rather than an extreme form of openness to experience or intellectual curiosity (40).

In different conceptualizations, several studies investigated the link between Machiavellianism and personality dysfunction. McHoskey (41) found that Machiavellianism was positively associated with an index of general personality dysfunction. At the level of specific PDs, he found that Machiavellianism was most strongly correlated with Borderline PD symptoms (41). Several authors (42–44) argued that Borderderline PD is not a specific PD but an underlying common factor for all PDs, reflecting severity rather than a specific cluster of PD symptoms. Therefore, Machiavellianism’s association with Borderline PD could also indicate the link between Machiavellianism and impairments of personality functioning in general. Láng (45, 46) found that higher levels of Machiavellianism were associated with more frequent interpersonal difficulties related to PDs and more intense presence of indices of Borderline Personality Organization – a dynamic object relations theory conceptualization of level of personality functioning (47). Similarly from a dynamic approach, Jauk and Ehrenthal (48) found that more impaired object-related (i.e., interpersonal) regulation was associated with higher levels of Machiavellianism. Investigating the relationship between Dark Tetrad (i.e., Machiavellianism, Psychopathy, Narcissism, and Sadism) and workplace outcomes as mediated by level of personality functioning, Zeigler-Hill and Besser (49) found that level of personality functioning’s three aspects out of four – impairments related to identity, empathy, and intimacy – were positively associated with Machiavellianism.

As pathological personality traits are maladaptive counterparts of dimensions from the five-factor model of personality (35, 50), we begin with an overview of studies that tested the associations between Machiavellianism and the Big Five. In general, lower levels of agreeableness and conscientiousness were repeatedly found to be associated with higher levels of Machiavellianism (51–53). Further, DeShong and colleagues (51) found that Machiavellianism was negatively associated with some facets of extraversion (e.g., warmth and activity) and positively with some facets of neuroticism (e.g., angry hostility and depressiveness). As for DSM-5 related pathological personality traits, Machiavellianism was consequently found to be positively correlated with Antagonism and Psychoticism domains (54–56). Further, Grigoras and Wille (54) found that higher levels of Machiavellianism were associated with higher levels of Negative Affectivity, while Qaderi Bagajan and colleagues (55) found all pathological personality domains to be positively associated with Machiavellianism.

Through a detailed investigation of maladaptive personality facets (i.e., different aspects of traits; 54; Wissing & Reinhard) – and in line with the Five Factor conceptualization of Machiavellianism (57, 58) –, Machiavellianism can be conceptualized in the AMPD as follows. The core manipulative and exploitative nature of Machiavellianism maps onto the maladaptive personality facets of Manipulativeness and Deceitfulness (35; both facets of Antagonism). The facets of Callousness (representing lack of empathy) and Anxiousness (low; representing emotional detachment) can be dispositions that help remaining emotionally detached during the act of manipulation – known as the Machiavellian cool syndrome – and avoiding the emotional costs (e.g., guilt; 46) of harming others. The facet of Rigid Perfectioninsm (low Disinhibition) can account for the strategic, long-term oriented nature of Machiavellian manipulation (59). Finally, Suspiciousness facet from Detachment maladaptive personality trait (35) could be a conceptual counterpart of Machiavellian views (1, 4) in representing a cynical and defensive attitude towards the world and other people.

As the above conceptual and empirically backed up conclusions come from a mainly unidimensional conceptualization of Machiavellianism, the risk that “opposing correlations between each subscale and a dimension of psychopathology would cause a null correlation” (2, p. 79.) is substantial. Therefore, some related evidence from research with two-dimensional conceptualizations should be presented. In exploring the link between the Dark Triad (i.e., Machiavellianism, Psychopathy, and Narcissism) and emotions, Davis (60) found that both Machiavellian views and tactics were negatively associated with CARE and positively with ANGER, while the negative association with PLAY (i.e., positive emotions) and the positive association with FEAR were only characteristic of Machiavellian views. Similarly, in exploring the role of Machiavellian views and tactics in psychopathology, Monaghan and colleagues (2) found that Machiavellian views were positively associated will all kind of psychopathologies (i.e., internalizing problems, externalizing problems, and thought disorder), Machiavellian tactics were only associated with externalizing problems.

Previous research in clinical psychology suggested that level of personality functioning could serve as a mediating variable between various predictor and outcome variables. For example, the mediating role of level of personality functioning was proven by several studies (61–64) for the relationship between childhood maltreatment and a variety of adult mental health outcomes (e.g., mental distress, somatoform complaints, anxiety, depression, and loneliness). Because the presence of maladaptive personality traits can add up to a symptom load that can impair personality functioning (65–67), the mediating effect of level of personality functioning between maladaptive personality trait domains and the two dimensions of Machiavellianism is also worth testing.

## 2 The current study

The aim of the current study was threefold. First, we wanted (a) to test whether previously established associations between pathological personality trait domains and Machiavellianism would apply to a clinical sample, and (b) to further our understanding of this connection with using a two-dimensional conceptualization of Machiavellianism. Based on previous research summarized in the Introduction, we expected that both dimensions of Machiavellianism would be positively correlated with each of the pathological personality traits (Hypothesis 1). For Antagonism and Disinhibition, we expected stronger correlations with Machiavellian tactics (as compared to Machiavellian views; Hypothesis 2). For Negative Affectivity and Detachment, we expected stronger correlations with Machiavellian views (as compared to Machiavellian tactics; Hypothesis 3).

Second, we expected that both dimensions of Machiavellianism would be positively correlated with impairments in personality functioning (Hypothesis 4). Third, we wanted to test the potential mediating role of level of personality functioning between pathological personality trait domains and the two dimensions of Machiavellianism. Because we were ignorant of any study testing this mediation model, this aim was completely exploratory.

## 3 Method

### 3.1 Procedure

As part of an ongoing data collection at an in-patient psychotherapy unit in the Hungarian capital, the measures for this study were completed by patients between 2021 February and 2023 March. After giving their informed consent (for ethical approval see Ethics Statement section), they anonymously participated in the study. Codes were used to match medical and demographical information with measurement data. Data collection took place as part of patients’ admission process. Questionnaires were filled out in paper pencil format. Participation was voluntary, no rewards were offered. Participation, withdrawal from or lack of it had no effect on patients’ access to treatment.

### 3.2 Sample

Exclusion criteria were only set for diagnoses (see next paragraph). Otherwise, consent was the only inclusion criterion. A total number of 341 patients participated in the study. 200 patients identified as females, 94 as males, one as non-binary. Forty-six participants didn’t report gender. With 48 participants not disclosing their age, mean age of the participants was 34.78 years (SD = 10.99), ranging from 21 to 73. Regarding educational level, 52 participants didn’t report, 253 patients at least graduated from secondary school, while the remaining 26 patients had vocational education or no secondary education. We examined the demographic data using the Little MCAR test (68). The results showed that there were multiple patterns of missing data (k=5) and the test didn’t reject the null hypothesis (χ²(5) = 1.27, p = .94), suggesting that the missing data can be considered completely at random.

As part of the admission process, patients were diagnosed using ICD-10 codes. Diagnoses were assigned by one of the psychiatrists from the staff. They were all blind to the goal of the current study. Schizophrenia, schizotypal and delusional disorders (F20-29)s and autism spectrum disorders (F84.x) were considered as exclusion criteria. With missing diagnosis for 45 patients, 146 received a primary diagnosis of Anxiety, dissociative, stress-related, somatoform and other nonpsychotic mental disorders (F40-48), 100 Mood disorders (F30-39), and 37 Disorders of adult personality and behavior (F60-69). The remaining thirteen patients received other, miscellaneous primary diagnoses.

Besides reporting demographic data (age, gender, level of education), all participants completed self-report measures of pathological personality traits, level of personality functioning, and Machiavellianism.

### 3.3 Measures

Personality Inventory for DSM-5 – Brief Form (35, 40 for the original version). The PID-5-BF is a 25-item self-report questionnaire to assess pathological personality traits in line with DSM-5 AMPD. Pathological personality domains are measured with five items each (e.g., ‘People would describe me as reckless’, ‘I often feel like nothing I do really matters’, ‘My thoughts often don’t make sense to others’, ‘I worry about almost everything’, and ‘It’s no big deal if I hurt other peoples’ feelings’ for Disinhibition, Detachment, Psychoticism, Negative Affectivity, and Antagonism, respectively). Items were rated on a 4-point Likert scale from 0 (completely untrue) to 3 (completely true). Domain scores were calculated by averaging item-level responses. Higher scores on each scale indicate the presence of more pronounced pathological personality traits in the given domain.

Hungarian version of Level of Personality Functioning Scale – Brief Form 2.0 (LPFS-BF 2.0 H; 26, 69 for the original version). The LPFS-BF 2.0 H is a 12-item self-report questionnaire to assess level of personality functioning in line with DSM-5 AMPD. Items (e.g., ‘I often do not know who I really am’) were rated on a 4-point Likert scale from 0 (completely untrue) to 3 (completely true). In this study, total score (average of twelve item-level responses) was used. Higher scores indicate more severe impairment of personality functioning.

Two-Dimensional Machiavellianism Scale (TDMS; 4). TDMS is a two-dimensional, 12-item measure of Machiavellianism. Six items measure Machiavellian Views (e.g., “In my opinion, human nature is to be dishonest”), the affective-cognitive domain of Machiavellianism. Six items measure Machiavellian Tactics (e.g., “I think that it is OK to be unethical for the greater good”), the cognitive-behavioral dimension of Machiavellianism. Participants rated items on a 7-point Likert scale from 1 (completely disagree) to 7 (completely agree). Item-level responses were averaged to form Machiavellian Views and Machiavellian Tactics scores. The Hungarian version was compiled using the translation-backtranslation method (70).

### 3.4 Statistical analytical plan

Jamovi 2.2 (71) was used to perform all the statistical analyses. Means, standard deviations, and McDonald’s ω values (72) were computed to describe measured variables and to test the internal reliability of scales, respectively The assumption of normality was not violated, the absolute values of Skewness and Kurtosis were less than two for all variables used (73).

The structural validity of TDMS was tested with Confirmatory Factor Analysis (CFA) with Diagonally Weighted Least Squares (DWLS) estimation method. To evaluate model fit, cutoffs suggested by (74) were used. CFI/TLI >= .95, RMSEA <= .06, and SRMR <= .08 were considered to indicate a good fit. Associations between measured variables were tested withPearson’s correlations. To compare strengths of correlations between pathological personality domains and Machiavellian views vs. pathological personality traits and Machiavellian tactics, we used r-to-z transformation. Two-tailed Z scores and their level of significance were calculated online (Eid et al., 2011 in 75).

“Although SEMs are known to be superior to path models because they take measurement errors into account, researchers can choose path mediation models instead of corresponding SEMs if insufficient sample sizes were inevitably collected” (76, p. 96). Because our sample size (all available patients in a time range of one and a half years) was insufficient for Structural Equation Models (SEMs; 76), the potential mediating effect of level of personality functioning between pathological traits and Machiavellianism was tested with two GLM mediation path analyses separately for Machiavellian Views and Machiavellian Tactics. In line with Zhao and colleagues’ (77) suggestion, indirect and direct effects are reported in order to sort mediation effects into five potential categories: (1) complementary mediation: both indirect and direct effects are significant and have the same direction; (2) competitive mediation: both indirect and direct effects are significant and have opposite directions; (3) indirect-only mediation: there is and indirect effect but no direct effect; (4) direct-only nonmediation: there is a direct effect but no indirect effect; (5) no-effect nonmediation: neither direct nor indirect effects are significant. Standardized estimates of both direct and indirect effects are presented alongside 95% confidence intervals obtained using 5,000 bootstrap resamples. Multicollinearity were not a concern in the models (VIF values were below 4).

## 4 Results

Means, standard deviations of the measured variables and internal reliability indices of scales are presented in **Table 1**. According to McDonald’s ω values, all scales reached an acceptable level (ω >.60) of internal consistency. Detachment, Negative Affectivity, and Psychoticism fell short of the common standard of ω >= .70. Before testing our hypotheses, the structural validity of the Hungarian translation of TDMS was tested. Results of CFA with DWLS estimation method indicated a good fit for the two correlated factors model without modification indices [CFI = .969; TLI = .960; RMSEA (90%CI) = .063 (.047 -.079); SRMR = .074; see **Supplementary Figure 1** for additional information].

**Table 1:** Descriptives and internal reliability of and associations between measured variables (Pearson’s correlations).

		TDMS		LPFS-BF	PID-5-BF domains				
		Views	Tactics		DIS	D	PSY	NA	ANT
TDMS Tactics		.368***							
LPFS-BF		.375***	.298***						
PID-5-BF Domains	DIS	.202***	.279***	.525***					
	D	.332***	.236***	.501***	.205***				
	PSY	.301***	.279***	.611***	.495***	.323***			
	NA	.192***	.037	.554***	.508***	.155**	.402***		
	ANT	.288***	.475***	.448***	.427***	.248***	.347***	.306***	
M		3.560	2.561	1.665	1.262	1.342	0.928	1.526	0.633
SD		1.073	1.188	0.575	0.781	0.697	0.709	0.681	0.521
Skewness		0.232	0.833	-0.832	0.243	0.033	0.631	-0.225	1.108
Kurtosis		-0.427	0.161	0.250	-0.869	-0.616	-0.103	-0.456	1.313
McDonald’s ω		.817	.870	.819	.833	.679	.785	.681	.708

PID-5-BF, Personality Inventory for DSM-5 – Brief Form; DIS, Disinhibition; D, Detachment; PSY, Psychoticism; NA, Negative Affectivity; ANT, Antagonism; TDMS, Two-Dimensional Machiavellianism Scale; LPFS-BF, Level of Personality Functioning Scale – Brief Form 2.0. ** p < 0.01; *** p < 0.001.

In Hypothesis 1, we expected all PID-5-BF scales to be significantly correlated with both TDMS scales. We used Pearson’s correlation to test this hypothesis. According to results presented in **Table 1** and in **Supplementary Figure 2**, all pathological personality domains were positively and significantly correlated with both TDMS Views and Tactics, except for Negative Affectivity. Negative Affectivity was not correlated with TDMS Tactics, while it was significantly correlated with TDMS Views. Thus, hypothesis 1 was only partially supported.

In testing Hypothesis 2, we used r-to-z transformation. For Disinhibition, results (z = -1.311; p = .095) showed that the correlation of Disinhibition with TDMS Tactics was stronger than its correlation with TDMS Views. Antagonism’s correlation with TDMS Tactics proved to be stronger than its correlation with TDMS Views (z = -3.421; p <.001). Thus, Hypothesis 2 was partially supported. In testing Hypothesis 3, we used the same method as for testing Hypothesis 2. According to results both Detachment (z = 1.662; p = .048) and Negative Affectivity (z = 2.562; p = .005) were more strongly correlated with TDMS Views than with TDMS Tactics. Thus, Hypothesis 3 was supported.

In Hypothesis 4, we expected both TDMS scales to be significantly correlated with level of personality functioning. According to results presented in **Table 1**, both correlations were positive and significant with a weak to moderate strength. This indicated that relatively more pronounced Machiavellian traits – both from cognitive-emotional and cognitive-behavioral aspects – were associated with relatively more impaired personality functioning. Thus, Hypothesis 4 was supported.

**Figure 1:**
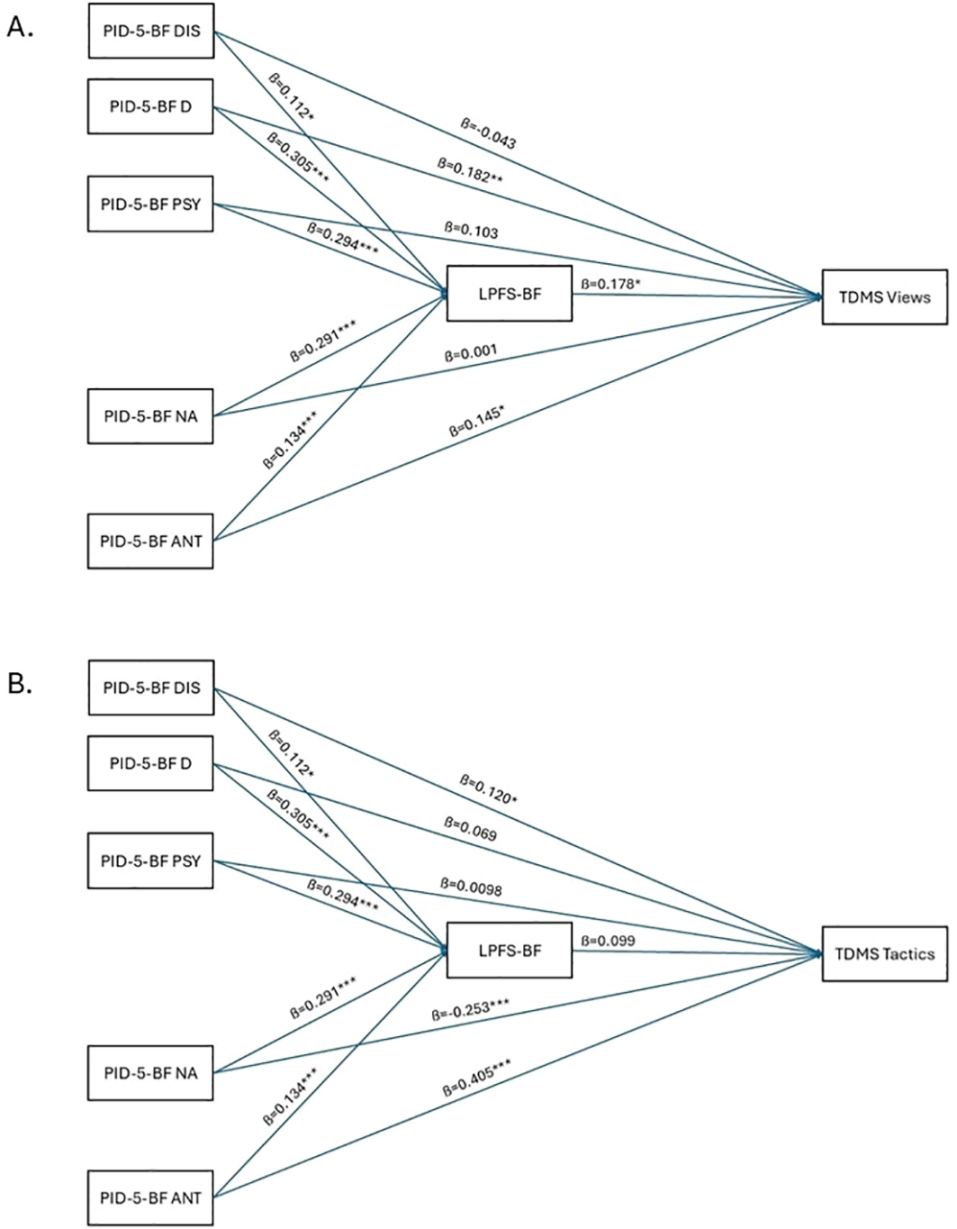
Path diagrams for the mediation models. Uppert part **(A)** presents the paths for the mediation model regarding TDMS Views, while the bottom part **(B)** presents the paths for the mediation model regarding TDMS Tactics. The reported estimates are standardized estimates (β). PID-5-BF, Personality Inventory for DSM-5 – Brief Form; DIS, Disinhibition; D, Detachment; PSY, Psychoticism; NA, Negative Affectivity; ANT, Antagonism; TDMS, Two Dimensional Machiavellianism Scale; LPFS-BF, Level of Personality Functioning Scale – Brief Form 2.0. *p < 0.05; **p < 0.01; ***p < 0.001.

As for the third, exploratory aim of our study, we tested whether level of personality functioning would mediate the association between pathological personality traits and dimensions of Machiavellianism. The explore this issue, two separate GLM mediation analyses were performed. Results of these analyses are presented in **Figure 1**, in **Table 2** and summarized in **Supplementary Table 1**.

**Table 2:** Indirect (through level of personality functioning) and direct effects of pathological personality traits on Machiavellian Views and Tactics; results of GLM mediation analyses.

Effect type	Effect	TDMS views				TDMS tactics			
		ß	CI 95%		p	ß	CI 95%		p
			Lower	Upper			Lower	Upper	
Indirect	PID-5-BF DIS ⇒ LPFS-BF ⇒ TDMS	0.020	0.003	0.071	0.089	0.011	-0.003	0.059	0.232
	PID-5-BF D ⇒ LPFS-BF ⇒ TDMS	**0.054**	**0.008**	**0.165**	**0.028**	0.030	-0.02	0.137	0.182
	PID-5-BF PSY ⇒ LPFS-BF ⇒ TDMS	**0.052**	**0.008**	**0.159**	**0.030**	0.029	-0.0186	0.132	0.185
	PID-5-BF NA ⇒ LPFS-BF ⇒ TDMS	**0.052**	**0.007**	**0.173**	**0.030**	0.029	-0.01799	0.134	0.184
	PID-5-BF ANT ⇒ LPFS-BF ⇒ TDMS	0.024	0.005	0.121	0.056	0.013	-0.008	0.098	0.208
Direct	PID-5-BF DIS ⇒ TDMS	-0.043	-0.24	0.124	0.504	0.120	0.009	0.376	0.044
	PID-5-BF D ⇒ TDMS	**0.182**	**0.101**	**0.45**	**0.002**	0.069	-0.066	0.301	0.198
	PID-5-BF PSY ⇒ TDMS	0.103	-0.055	0.343	0.105	0.098	-0.071	0.393	0.101
	PID-5-BF NA ⇒ TDMS	0.001	-0.212	0.212	0.993	**-0.253**	**-0.628**	**-0.258**	**< .001**
	PID-5-BF ANT ⇒ TDMS	**0.145**	**0.039**	**0.568**	**0.010**	**0.405**	**0.63**	**1.236**	**< .001**
Component	LPFS-BF ⇒ TDMS	**0.178**	**0.021**	**0.636**	**0.023**	0.099	-0.088	0.507	0.177
	PID-5-BF DIS ⇒ LPFS-BF	**0.112**	**0.021**	**0.147**	**< .001**	0.112	0.023	0.145	0.10
	PID-5-BF D ⇒ LPFS-BF	**0.305**	**0.192**	**0.311**	**< .001**	**0.305**	**0.190**	**0.310**	**< .001**
	PID-5-BF PSY ⇒ LPFS-BF	**0.294**	**0.176**	**0.300**	**< .001**	**0.294**	**0.175**	**0.301**	**< .001**
	PID-5-BF NA ⇒ LPFS-BF	**0.291**	**0.13**	**0.319**	**< .001**	**0.291**	**0.176**	**0.318**	**< .001**
	PID-5-BF ANT ⇒ LPFS-BF	**0.134**	**0.06**	**0.238**	**< .001**	**0.134**	**0.059**	**0.236**	**< .001**
Total	PID-5-BF DIS ⇒ TDMS	-0.0226	-0.211	0.159	0.723	**0.131**	**0.029**	**0.377**	**0.027**
	PID-5-BF D ⇒ TDMS	**0.236**	**0.2**	**0.514**	**< .001**	**0.099**	**0.004**	**0.332**	**0.043**
	PID-5-BF PSY ⇒ TDMS	**0.156**	**0.055**	**0.419**	**0.010**	**0.127**	**-0.003**	**0.429**	**0.023**
	PID-5-BF NA ⇒ TDMS	0.052	-0.105	0.269	0.370	**-0.224**	**-0.569**	**-0.221**	**< .001**
	PID-5-BF ANT ⇒ TDMS	**0.169**	**0.092**	**0.608**	**0.002**	**0.418**	**0.658**	**1.246**	**< .001**
	TDMS Views (R^2^)				TDMS Tactics (R^2^)				
Total effect	0.179				0.286				
Mediator model	0613				0.613				
Full model effect	0.192				0.290				

The presented values are standardized estimates (β) and the 95% confidence interval with p values.

PID-5-BF, Personality Inventory for DSM-5 – Brief Form; DIS, Disinhibition; D, Detachment; PSY, Psychoticism; NA, Negative Affectivity; ANT, Antagonism; TDMS, Two-Dimensional Machiavellianism Scale; LPFS-BF, Level of Personality Functioning Scale – Brief Form 2.0. Significant results are highlighted in bold.

The first GLM mediation analysis predicting levels of TDMS Views from maladaptive personality traits mediated by level of personality functioning revealed the following results. Disinhibition predicted TDMS Views neither directly nor indirectly. Higher levels of Detachment predicted higher levels of TDMS Views both directly and indirectly (mediated by level of personality functioning). This means that more pronounced social and emotional withdrawal predicted more pronounced cynical and untrustful attitude towards the world and others both through and independent from level of personality functioning. Higher levels of Psychoticism and Negative Affectivity predicted higher levels of TDMS Views only indirectly (mediated by level of personality functioning). This means that more pronounced peculiar thoughts and eccentric behavior, and more intense depression, anxiety, and anger predicted more pronounced cynical and untrustful attitude towards the world and others in a way that higher levels of these maladaptive traits predicted more impairment in personality functioning that in turn predicted more pronounced cynical stance towards the world and others. Lastly, higher levels of Antagonism only directly predicted higher levels of TDMS Views. This means that more pronounced coercive attitudes predicted more pronounced cynical and untrustful attitude towards the world and others independent from level of personality functioning.

In the second GLM mediation analysis predicting levels of TDMS Tactics by maladaptive personality traits mediated by level of personality functioning, the following results emerged. None of the indirect pathways – from maladaptive personality traits to TDMS Tactics through level of personality functioning – proved to be significant. At the same time, lower levels of Negative Affectivity and higher levels of Antagonism directly predicted higher levels of TDMS Tactics. All other maladaptive personality traits were unrelated to TDMS Tactics. This means that less pronounced self-reported presence of depressive, anxious and angry symptoms and a more intense presence of coercive attitude predicted a more pronounced willingness to exploit and manipulate others to achieve their goals, independent from level of personality functioning.

## 5 Discussion

To our knowledge, this study was the first that investigated the relationship between maladaptive personality trait domains and the two-dimensional conceptualization of Machiavellianism in a clinical sample using level of personality functioning as a potential mediator between the two set of constructs. The first aim of our study was to test the associations between pathological personality trait domains and Machiavellian views and tactics. All pathological personality trait domains were significantly and positively associated with both Machiavellian views and tactics, except for Negative Affectivity that was uncorrelated with Machiavellian tactics. These results partially confirmed Hypothesis 1 and are in line with the findings of DeShong and colleagues (51) who reported Machiavellianism to be associated with all dimensions of the five-factor model of personality except for Openness. Further, Qaderi Bagajan and colleagues (55) also found that all five pathological personality trait domains were positively associated with Machiavellianism. These results could indicate a high symptom load associated with Machiavellianism, similarly to the schema overflow present in Machiavellianism (78). Further results partially confirmed Hypotheses 2 and 3. Antagonism were more strongly associated with Machiavellian tactics, while Detachment and Negative Affectivity were more strongly associated with Machiavellian views. These results replicate earlier findings, where Machiavellian views were associated with internalizing and thought disorder domains and Machiavellian tactics were related to externalizing domains – let it be mental health symptoms (2) or emotions (60).

Regarding the second aim of our study, positive correlations were found between impairments in level of personality functioning and both dimensions of Machiavellianism. This confirmed Hypothesis 4. These results show that Machiavellianism is associated with a general dysfunction of personality as evidenced by previous studies by McHoskey (41)Láng (46), and Zeigler-Hill and Besser (49). They all found that higher levels of Machiavellianism were associated with more severe impairments in personality functioning – irrespective of the method used to assess personality dysfunction.

As for the exploratory aim of our study, we tested potential indirect pathways from maladaptive personality traits to dimensions of Machiavellianism via level of personality functioning. Higher levels of Machiavellian views were associated directly with higher levels of Detachment and Antagonism, while more intense presence of Detachment, Psychoticism, and Negative Affectivity were associated with more pronounced Machiavellian views via the indirect path through level of personality functioning. Higher levels of Machiavellian tactics were associated only directly with pathological personality traits in the model. More pronounced Antagonism and less pronounced Negative Affectivity were directly associated with higher levels of Machiavellian tactics. In the models, only Antagonism was found to be a associated with both dimensions of Machiavellianism, which is in line with the reliable emergence of disagreeableness as a common core not only to Machiavellianism but to all three Dark Triad personality traits (2, 53, 79, 80). The positive indirect associations with Detachment, Psychoticism, and Negative Affectivity through level of personality functioning show that these traits are associated with Machiavellian views at least in part (as for the case of Detachment) because they are associated with higher levels of personality dysfunction. Social withdrawal, peculiar thoughts, depressive and anxious symptoms can undermine level of personality functioning (81–83), which could lead to a cynical and untrustful view of the world and others where empathy and intimacy are rather dangerous. Although speculative in the current model, but Machiavellian views are easy to imagine to further deteriorate personality functioning leading to a vicious circle. Beyond the common association with higher levels of Antagonism, higher levels of Machiavellian tactics were associated with less intense presence of Negative Affectivity. The relative lack of depressive mood, anxiety and anger might contribute to the agentic aspect of Machiavellianism (84) that was originally termed as the Machiavellian cool syndrome by Christie & Geis (1).

Despite the novel results, some limitations of the study should be mentioned before discussing the results. First, despite the tempting mediational models, our study was a cross-sectional one that prevented us from drawing any causal conclusions, even if language might reflect such conclusions. Moreover, the size of the available sample prevented us from using SEMs. In the future, more advanced statistics (i.e., including measurement models) should be used with sufficient participants. Second, our study relied solely on self-report data. Self-perception accuracy – especially in a clinical sample – might be a crucial point of distortion. Third, both LPFS and PID-5-BF are brief measures of level of personality functioning and maladaptive personality traits. Therefore, they give only a low-resolution picture of associations with Machiavellianism. Future research in this topic should use longer measures to enable the investigation of associations at a facet level. Fourth, some of the PID-5-BF scales had lower internal consistency than expected. This might limit the reliability of our results, even if the broadness and brevity of the scales could be sufficient explanations for this shortcoming (85). In the future, multi-informant longitudinal research could make good for these limitations.

## 6 Conclusions

To conclude from a clinical point of view, PDs’ potential association with manipulation (e.g., 86, 87) and cynicism (e.g., 88) could be highlighted. Whereas cynicism could indicate a general decline in resilience (42; for the relationship between cynicism and burnout see 89), interpersonal manipulation and exploitation could serve as a defense mechanism against the cynically perceived world and mistrustful others (78). Moreover, conceptualizing patient personality in terms of the two dimensions of Machiavellianism (4) can guide clinicians in choosing best fitting screening measures to assess deviant expressions in personality. Patients with more prominent cynicism should be assessed for level of personality functioning (i.e., problematic functioning in domains like identity, self-directedness, empathy, and intimacy), while patients with more prominent manipulative tendencies should be assessed for maladaptive personality traits.

The association with three out of five maladaptive personality traits on Machiavellian Views (vs. none for Machiavellian Tactics) via the indirect pathway through level of personality functioning might suggest that Machiavellian views could account for the vulnerable side of Machiavellianism (90), whereas Machiavellian tactics (with no association with negative affectivity and a stronger association with antagonism – as compared to Machiavellian views) could represent the agentic side of Machiavellianism (84). Future research should test whether this hypothesized distinction between vulnerable and agentic Machiavellianism could help disentangle the overlapping constructs of Machiavellianism and psychopathy (91).

## Data Availability

The raw data supporting the conclusions of this article will be made available by the authors, without undue reservation.

## Footnotes

Please note that referring to this conceptualization ’pathological’ and ’maladaptive’ are used interchangeably.

## References

[1] Christie R Geis FL . Studies in Machiavellianism. Christie R Geis FL , editors. New York, NY: Academic Press (1970). doi: 10.1016/B978-0-12-174450-2.50001-4, PMID:

[2] Monaghan C Bizumic B Sellbom M . The role of Machiavellian views and tactics in psychopathology. Pers Individ Dif. (2016) 94:72–81. doi: 10.1016/j.paid.2016.01.002. PMID: 41936479

[3] Monaghan C Bizumic B Sellbom M . Nomological network of two-dimensional Machiavellianism. Pers Individ Dif. (2018) 130:161–73. doi: 10.1016/j.paid.2018.03.047. PMID: 41936479

[4] Monaghan C Bizumic B Williams T Sellbom M . Two-dimensional Machiavellianism: Conceptualization, theory, and measurement of the views and tactics dimensions. psychol Assess. (2020) 32:277–93. doi: 10.1037/pas0000784. PMID: 31750680

[5] Clark LA . Wherefrom and whither PD? Recent developments and future possibilities in DSM-5 and ICD-11 personality disorder diagnosis. Curr Psychiatry Rep. (2025) 27:267–77. doi: 10.1007/s11920-025-01602-y. PMID: 40108080 PMC12003573

[6] American Psychiatric Association . The Diagnostic and Statistical Manual of Mental Disorders, Fifth Edition. Arlington, VA: American Psychiatric Publishing (2013).

[7] World Health Organization . ICD-10: International statistical classification of diseases and related health problems: Tenth revision, 2nd ed. Geneva, Switzerland: World Health Organization (2004).

[8] World Helath Organization . ICD-11: International classification of diseases (11th revision). Geneva, Switzerland: World Helath Organization (2022).

[9] Bach B Kramer U Doering S Di Giacomo E Hutsebaut J Kaera A . The ICD-11 classification of personality disorders: A European perspective on challenges and opportunities. Borderline Pers Disord Emotion Dysregulation. (2022) 9:12. doi: 10.1186/s40479-022-00182-0. PMID: 35361271 PMC8973542

[10] Swales MA . Personality disorder diagnoses in ICD-11: Transforming conceptualisations and practice. Clin Psychol Europe. (2022) 4:e9635. doi: 10.32872/cpe.9635. PMID: 36760321 PMC9881116

[11] Jablensky A . The classification of personality disorders: Critical review and need for rethinking. Psychopathology. (2002) 35:112–6. doi: 10.1159/000065129. PMID: 12145494

[12] Kernberg OF . Overview and critique of the classification of personality disorders proposed for DSM-V. Schweizer Archiv Fr Neurol Und Psychiatr. (2012) 163:234–8. doi: 10.4414/sanp.2012.00110. PMID: 40251714

[13] Trull TJ Durrett CA . Categorical and dimensional models of personality disorder. Annu Rev Clin Psychol. (2005) 1:355–80. doi: 10.1146/annurev.clinpsy.1.102803.144009. PMID: 17716092

[14] Skodol AE Clark LA Bender DS Krueger RF Morey LC Verheul R . Proposed changes in personality and personality disorder assessment and diagnosis for DSM-5 part I: Description and rationale. Pers Disorders: Theory Research Treat. (2011) 2:4–22. doi: 10.1037/a0021891. PMID: 22448687

[15] Hopwood CJ Kotov R Krueger RF Watson D Widiger TA Althoff RR . The time has come for dimensional personality disorder diagnosis. Pers Ment Health. (2018) 12:82–6. doi: 10.1002/pmh.1408. PMID: 29226598 PMC5811364

[16] Widiger TA Hines A . The Diagnostic and Statistical Manual of Mental Disorders, Fifth Edition alternative model of personality disorder. Pers Disorders: Theory Research Treat. (2022) 13:347–55. doi: 10.1037/per0000524. PMID: 35787119

[17] Bogaerts A Luyckx K Bastiaens T Kaufman EA Claes L . Identity impairment as a central dimension in personality pathology. J Psychopathol Behav Assess. (2021) 43:33–42. doi: 10.1007/s10862-020-09804-9. PMID: 41933263

[18] Bogaerts A Luyckx K Bastiaens T Sleuwaegen E Berens A Claes L . The self-concept and identity measure in patients with personality disorders: A psychometric evaluation and associations with identity processes, core domains of self-functioning, and personality disorder symptoms. Assessment. (2023) 30:2184–97. doi: 10.1177/10731911221140313. PMID: 36594676

[19] Few LR Miller JD Rothbaum AO Meller S Maples J Terry DP . Examination of the section III DSM-5 diagnostic system for personality disorders in an outpatient clinical sample. J Abnormal Psychol. (2013) 122:1057–69. doi: 10.1037/a0034878. PMID: 24364607 PMC4105005

[20] Hentschel AG John Livesley W . Differentiating normal and disordered personality using the general assessment of personality disorder (GAPD). Pers Ment Health. (2013) 7:133–42. doi: 10.1002/pmh.1218. PMID: 24343939

[21] Hörz-Sagstetter S Ohse L Kampe L . Three dimensional approaches to personality disorders: A review on personality functioning, personality structure, and personality organization. Curr Psychiatry Rep. (2021) 23:45. doi: 10.1007/s11920-021-01250-y. PMID: 34181116 PMC8238706

[22] Sharp C . Adolescent personality pathology and the alternative model for personality disorders: Self development as nexus. Psychopathology. (2020) 53:198–204. doi: 10.1159/000507588. PMID: 32464626

[23] Sharp C Wall K . DSM-5 level of personality functioning: Refocusing personality disorder on what it means to be human. Annu Rev Clin Psychol. (2021) 17:313–37. doi: 10.1146/annurev-clinpsy-081219-105402. PMID: 33306924

[24] Erkoreka L Zamalloa I Rodriguez S Muñoz P Mendizabal I Zamalloa MI . Attachment anxiety as mediator of the relationship between childhood trauma and personality dysfunction in borderline personality disorder. Clin Psychol Psychother. (2022) 29:501–11. doi: 10.1002/cpp.2640. PMID: 34228846

[25] Jeung H Herpertz SC . Impairments of interpersonal functioning: Empathy and intimacy in borderline personality disorder. Psychopathology. (2014) 47:220–34. doi: 10.1159/000357191. PMID: 24577235

[26] Láng A Birkás B . [Psychometric poperties of the Hungarian version of Level of Personality Functioning – Brief Form 2.0 (LPFS-BF 2.0 H)]. (2023) 24:100–112. doi: 10.1556/0406.2023.00031.

[27] Luyten P Campbell C Fonagy P . Rethinking the relationship between attachment and personality disorder. Curr Opin Psychology Pers Pathology: Dev Aspects. (2021) 37:109–13. doi: 10.1016/j.copsyc.2020.11.003. PMID: 33385979

[28] Salgado RM Pedrosa R Bastos-Leite AJ . Dysfunction of empathy and related processes in borderline personality disorder: A systematic review. Harvard Rev Psychiatry. (2020) 28:238. doi: 10.1097/HRP.0000000000000260. PMID: 32692088 PMC7357542

[29] Siefert CJ Sexton J Meehan K Nelson S Haggerty G Dauphin B . Development of a short form for the DSM–5 levels of personality functioning questionnaire. J Pers Assess. (2020) 102:516–26. doi: 10.1080/00223891.2019.1594842. PMID: 31107606

[30] Wilson S Stroud CB Durbin CE . Interpersonal dysfunction in personality disorders: A meta-analytic review. psychol Bull. (2017) 143:677–734. doi: 10.1037/bul0000101. PMID: 28447827 PMC5507693

[31] Hopwood CJ . Personality functioning, problems in living, and personality traits. J Pers Assess. (2025) 107:143–58. doi: 10.1080/00223891.2024.2345880. PMID: 38700238

[32] Birkhölzer M Schmeck K Goth K . Assessment of criterion a. Curr Opin Psychology Pers Pathology: Dev Aspects. (2021) 37:98–103. doi: 10.1016/j.copsyc.2020.09.009. PMID: 33099168

[33] Krueger RF Hobbs KA . An overview of the DSM-5 alternative model of personality disorders. Psychopathology. (2020) 53:126–32. doi: 10.1159/000508538. PMID: 32645701 PMC7529724

[34] Zimmermann J Kerber A Rek K Hopwood CJ Krueger RF . A brief but comprehensive review of research on the alternative DSM-5 model for personality disorders. Curr Psychiatry Rep. (2019) 21:92. doi: 10.1007/s11920-019-1079-z. PMID: 31410586

[35] Krueger RF Derringer J Markon KE Watson D Skodol AE . Initial construction of a maladaptive personality trait model and inventory for DSM-5. Psychol Med. (2012) 42:1879–90. doi: 10.1017/S0033291711002674. PMID: 22153017 PMC3413381

[36] Griffin SA Samuel DB . A closer look at the lower-order structure of the personality inventory for DSM-5: Comparison with the five-factor model. Pers Disorders: Theory Research Treat. (2014) 5:406–12. doi: 10.1037/per0000074. PMID: 24886053

[37] Suzuki T Samuel DB Pahlen S Krueger RF . DSM-5 alternative personality disorder model traits as maladaptive extreme variants of the five-factor model: An item-response theory analysis. J Abnormal Psychol. (2015) 124:343–54. doi: 10.1037/abn0000035. PMID: 25665165

[38] Watson D Stasik SM Ro E Clark LA . Integrating normal and pathological personality: Relating the DSM-5 trait-dimensional model to general traits of personality. Assessment. (2013) 20:312–26. doi: 10.1177/1073191113485810. PMID: 23596272

[39] Chmielewski M Bagby RM Markon K Ring AJ Ryder AG . Openness to experience, intellect, schizotypal personality disorder, and psychoticism: Resolving the controversy. J Pers Disord. (2014) 28:483–99. doi: 10.1521/pedi_2014_28_128. PMID: 24511900

[40] Birkás B Kállai J Hupuczi E Bandi SA Láng A . Experiences with the validation of the Hungarian version of Personality Inventory for DSM-5 Brief Form: Predicting personality disorders based on self-report inventory. Psychiatria Hungarica: A Magyar Pszichiatriai Tarsasag Tudomanyos Folyoirata. (2018) 33:270–81. 30426933

[41] McHoskey JW . Machiavellianism and personality dysfunction. Pers Individ Dif. (2001) 31:791–8. doi: 10.1016/s0191-8869(00)00187-2. PMID: 41334505

[42] Fonagy P Luyten P Allison E Campbell C . What we have changed our minds about: Part 1. Borderline personality disorder as a limitation of resilience. Borderline Pers Disord Emotion Dysregulation. (2017) 4. doi: 10.1186/s40479-017-0061-9. PMID: 28413687 PMC5389119

[43] Leichsenring F Fonagy P Heim N Kernberg OF Leweke F Luyten P . Borderline personality disorder: A comprehensive review of diagnosis and clinical presentation, etiology, treatment, and current controversies. World Psychiatry. (2024) 23:4–25. doi: 10.1002/wps.21156. PMID: 38214629 PMC10786009

[44] Sharp C Wright AGC Fowler JC Frueh BC Allen JG Oldham J . The structure of personality pathology: Both general (‘g’) and specific (‘s’) factors? J Abnormal Psychol. (2015) 124:387–98. doi: 10.1037/abn0000033. PMID: 25730515

[45] Láng A . Machiavellianism and personality disorder: Their relationship in the mirror of interpersonal attitudes. Orvosi Hetilap. (2014) 155:1584–8. doi: 10.1556/oh.2014.30004. PMID: 25261989

[46] Láng A . Borderline personality organization predicts Machiavellian interpersonal tactics. Pers Individ Dif. (2015) 80:28–31. doi: 10.1016/j.paid.2015.02.022. PMID: 41936479

[47] Clarkin JF Lenzenweger MF Yeomans F Levy KN Kernberg OF . An object relations model of borderline pathology. J Pers Disord. (2007) 21:474–99. doi: 10.1521/pedi.2007.21.5.474. PMID: 17953502

[48] Jauk E Ehrenthal JC . Self-reported levels of personality functioning from the operationalized psychodynamic diagnosis (OPD) system and emotional intelligence likely assess the same latent construct. J Pers Assess. (2021) 103:365–79. doi: 10.1080/00223891.2020.1775089. PMID: 32631173 PMC7611281

[49] Zeigler-Hill V Besser A . Dark personality features and workplace outcomes: The mediating role of difficulties in personality functioning. Curr Psychol. (2021) 40:5430–44. doi: 10.1007/s12144-019-00527-z. PMID: 41933263

[50] Garofalo C Virgilio C Bogaerts S Schimmenti A . Dark ladies: Maladaptive personality domains, alexithymia, and the dark triad in women. Clin Neuropsychiatry. (2020) 16:221–8. doi: 10.36131/clinicalnpsych2019050605. PMID: 34908959 PMC8650191

[51] DeShong HL Helle AC Lengel GJ Meyer N Mullins-Sweatt SN . Facets of the dark triad: Utilizing the five-factor model to describe Machiavellianism. Pers Individ Dif. (2017) 105:218–23. doi: 10.1016/j.paid.2016.09.053. PMID: 41936479

[52] Lee K Ashton MC . Psychopathy, Machiavellianism, and narcissism in the five-factor model and the HEXACO model of personality structure. Pers Individ Dif. (2005) 38:1571–82. doi: 10.1016/j.paid.2004.09.016. PMID: 41936479

[53] Paulhus DL Williams KM . The dark triad of personality: Narcissism, Machiavellianism, and psychopathy. J Res Pers. (2002) 36:556–63. doi: 10.1016/s0092-6566(02)00505-6. PMID: 41881759

[54] Grigoras M Wille B . Shedding light on the dark side: Associations between the dark triad and the DSM-5 maladaptive trait model. Pers Individ Dif. (2017) 104:516–21. doi: 10.1016/j.paid.2016.09.016. PMID: 41936479

[55] Qaderi Bagajan K Ziegler M Soleimani M Paulhus DL Soleimani ZA Kordbagheri M . Validation of the Short Dark Tetrad (SD4) in Persian: Assessment of its structure and nomological network. J Individ Dif. (2024) 45:135–47. doi: 10.1027/1614-0001/a000417, PMID: 37214235

[56] Wissing BG Reinhard M-A . The dark triad and the PID-5 maladaptive personality traits: Accuracy, confidence and response bias in judgments of veracity. Front Psychol. (2017) 8:1549. doi: 10.3389/fpsyg.2017.01549. PMID: 28983264 PMC5613765

[57] Collison KL Vize CE Miller JD Lynam DR . Development and preliminary validation of a five factor model measure of Machiavellianism. psychol Assess. (2018) 30:1401–7. doi: 10.1037/pas0000637. PMID: 30047746

[58] Kückelhaus BP Blickle G Kranefeld I Körnig T Genau HA . Five factor Machiavellianism: Validation of a new measure. J Pers Assess. (2021) 103:509–22. doi: 10.1080/00223891.2020.1784182. PMID: 32633560

[59] Jones DN Paulhus DL . Introducing the short dark triad (SD3): A brief measure of dark personality traits. Assessment. (2014) 21:28–41. doi: 10.1177/1073191113514105. PMID: 24322012

[60] Davis KR . EXPLORING THE RELATIONSHSIPS AMONG DARK PERSONALITY AND BASIC AFFECTIVE TRAITS. Boone, NC: Appalachian State University (2022).

[61] Freier A Kruse J Schmalbach B Zara S Werner S Brähler E . The mediation effect of personality functioning between different types of child maltreatment and the development of depression/anxiety symptoms – A German representative study. J Affect Disord. (2022) 299:408–15. doi: 10.1016/j.jad.2021.12.020. PMID: 34906643

[62] Krakau L Tibubos AN Beutel ME Ehrenthal JC Gieler U Brähler E . Personality functioning as a mediator of adult mental health following child maltreatment. J Affect Disord. (2021) 291:126–34. doi: 10.1016/j.jad.2021.05.006. PMID: 34034217

[63] Maerz J Viviani R Labek K . The key role of personality functioning in understanding the link between adverse childhood experiences and loneliness: A cross-sectional mediation analysis. Brain Sci. (2025) 15:6. doi: 10.3390/brainsci15060551. PMID: 40563722 PMC12191166

[64] Vaccarezza S Opazo S Mundt AP Cortázar A Errázuriz P . Childhood maltreatment and depression in adult patients: The mediator role of personality functioning. Counselling Psychother Res. (2025) 25:e12866. doi: 10.1002/capr.12866. PMID: 41925065

[65] Bach B Hutsebaut J . Level of personality functioning scale–brief form 2.0: Utility in capturing personality problems in psychiatric outpatients and incarcerated addicts. J Pers Assess. (2018) 100:660–70. doi: 10.1080/00223891.2018.1428984. PMID: 29494782

[66] Mazreku G Birkhölzer M Cosgun S Kerber A Schmeck K Goth K . Impaired personality functioning in children and adolescents assessed with the LoPF-Q 6–18 PR in parent-report and convergence with maladaptive personality traits and personality structure in school and clinic samples. Children. (2023) 10:1186. doi: 10.3390/children10071186. PMID: 37508683 PMC10378110

[67] Sharp C Cervantes BR . Maladaptive self- and interpersonal functioning increments general psychiatric severity in the association with adolescent personality pathology. Children. (2023) 10:120. doi: 10.3390/children10010120. PMID: 36670670 PMC9856791

[68] Little RJ . A test of missing completely at random for multivariate data with missing values. Journal of the American statistical Association. (1988) 83(404):1198–1202.

[69] Weekers LC Hutsebaut J Kamphuis JH . The Level of Personality Functioning Scale-Brief Form 2.0: Update of a brief instrument for assessing level of personality functioning. Pers Ment Health. (2019) 13:3–14. doi: 10.1002/pmh.1434. PMID: 30230242

[70] Brislin RW . Back-translation for cross-cultural research. J Cross-Cultural Psychol. (1970) 1:185–216. doi: 10.1177/135910457000100301. PMID: 41930703

[71] The jamovi project . jamovi—Open statistical software for the desktop and cloud (2024). Available online at: https://www.jamovi.org/ (Accessed April 28, 2025).

[72] Dunn TJ Baguley T Brunsden V . From alpha to omega: A practical solution to the pervasive problem of internal consistency estimation. Br J Psychol (London England: 1953). (2014) 105:399–412. doi: 10.1111/bjop.12046. PMID: 24844115

[73] George D Mallery P . IBM SPSS Statistics 26 Step by Step: A Simple Guide and Reference. New York, NY: Routledge (2019). doi: 10.4324/9780429056765

[74] Hu L Bentler PM . Cutoff criteria for fit indexes in covariance structure analysis: Conventional criteria versus new alternatives. Struct Equation Modeling. (1999) 6:1–55. doi: 10.1080/10705519909540118. PMID: 41909888

[75] Lenhard W Lenhard A . Hypothesis tests for comparing correlations. (2014). doi: 10.13140/RG.2.1.2954.1367

[76] Sim M Kim S-Y Suh Y . Sample size requirements for simple and complex mediation models. Educ psychol Measurement. (2022) 82:76–106. doi: 10.1177/00131644211003261. PMID: 34992307 PMC8725051

[77] Zhao X Lynch JG Chen Q . Reconsidering Baron and Kenny: Myths and truths about mediation analysis. J Consumer Res. (2010) 37:197–206. doi: 10.1086/651257, PMID: 40147090

[78] Láng A . Machiavellianism and early maladaptive schemas in adolescents. Pers Individ Dif. (2015) 87:162–5. doi: 10.1016/j.paid.2015.07.039. PMID: 41936479

[79] Moshagen M Zettler I Horsten LK Hilbig BE . Agreeableness and the common core of dark traits are functionally different constructs. J Res Pers. (2020) 87:103986. doi: 10.1016/j.jrp.2020.103986. PMID: 41936479

[80] Stead R Fekken GC . Agreeableness at the core of the dark triad of personality. Individ Dif Res. (2014) 12:131–41. doi: 10.65030/idr.12013

[81] Franquillo AC Guccione C Angelini G Carpentieri R Ducci G Caretti V . The role of personality in schizophrenia and psychosis: A systematic review. Clin Neuropsychiatry. (2021) 18:28–40. doi: 10.36131/cnfioritieditore20210103. PMID: 34909018 PMC8629049

[82] Jańczak MO Soroko E . Level of personality functioning and maladaptive personality traits in relation to depression and anxiety symptoms in middle and older adults. Sci Rep. (2025) 15, 11303. doi: 10.1038/s41598-025-96067-7. PMID: 40175487 PMC11965400

[83] Wagner-Skacel J Matzer F Kohlhammer-Dohr A Dalkner N Jauk E . Assessment of personality functioning in psychosomatic medicine. Wiener Klinische Wochenschrift. (2022) 134:602–10. doi: 10.1007/s00508-021-01993-x. PMID: 35344100 PMC9418278

[84] Jonason PK Fletcher SA . Agentic and communal behavioral biases in the dark triad traits. Pers Individ Dif. (2018) 130:76–82. doi: 10.1016/j.paid.2018.03.044. PMID: 41936479

[85] Gosling SD Rentfrow PJ Swann WB . A very brief measure of the big-five personality domains. J Res Pers. (2003) 37:504–28. doi: 10.1016/s0092-6566(03)00046-1. PMID: 41907667

[86] Mandal E Kocur D . Psychological masculinity, femininity and tactics of manipulation in patients with borderline personality disorder. Arch Psychiatry Psychother. (2013) 1:45–53.

[87] Mandal E Kocur D . The Machiavellianism and manipulation tactics used by patients with borderline personality disorder in everyday life and in therapy. Psychiatria Polska. (2013) 47:667–78. 24946473

[88] Frumer DS Ilan SD Fishman Y Weinberger R Gothelf D . Cynicism—a commonly used concept with relevance to mental health. Israel J Psychiatry. (2019) 56:3–10.

[89] Viljoen M Claassen N . Cynicism as subscale of burnout. Work. (2017) 56:499–503. doi: 10.3233/WOR-172518. PMID: 28339417

[90] Czibor A Szabo ZP Jones DN Zsido AN Paal T Szijjarto L . Male and female face of Machiavellianism: Opportunism or anxiety? Pers Individ Dif. (2017) 117:221–9. doi: 10.1016/j.paid.2017.06.002. PMID: 41936479

[91] Miller JD Hyatt CS Maples-Keller JL Carter NT Lynam DR . Psychopathy and Machiavellianism: A distinction without a difference? J Pers. (2017) 85:439–53. doi: 10.1111/jopy.12251. PMID: 26971566

