# Supplementary material for: Machiavellianism, level of personality functioning, and maladaptive personality traits: mediation analyses in a clinical sample
Source: Front Psychiatry. 2026 Apr 30;17:1675044. doi: 10.3389/fpsyt.2026.1675044 (PMC13171586; doi:10.3389/fpsyt.2026.1675044)
Supplement: Supplementary file 1 [file Table1.pdf]

Supplement Table 1. Summary of mediation results.

PID-5-BF Domain	Type of Effect	TDMS Views		TDMS Tactics	
		p<.05 (yes or no)	Type of Mediation Effect	p<.05 (yes or no)	Type of Mediation Effect
DIS	Indirect (through LPFS-BF)	no	No-effect (Nonmediation)	no	No-effect (Nonmediation)
	Direct	no		no	
D	Indirect (through LPFS-BF)	yes	**Complementary (Mediation)**	no	No-effect (Nonmediation)
	Direct	yes		no	
PSY	Indirect (through LPFS-BF)	yes	**Indirect-only (Mediation)**	no	No-effect (Nonmediation)
	Direct	no		no	
NA	Indirect (through LPFS-BF)	yes	**Indirect-only (Mediation)**	no	**Direct-only (Nonmediation)**
	Direct	no		yes	
ANT	Indirect (through LPFS-BF)	no	**Direct-only (Nonmediation)**	no	**Direct-only (Nonmediation)**
	Direct	yes		yes	

Note: PID-5-BF = Personality Inventory for DSM-5 – Brief Form; DIS = Disinhibition; D = Detachment; PSY = Psychoticism; NA = Negative Affectivity; ANT = Antagonism; TDMS = Two-Dimensional Machiavellianism Scale; LPFS-BF = Level of Personality Functioning Scale – Brief Form 2.0. Presence of any type of mediation effect is indicated in bold.
