# Supplementary material for: Machiavellianism, level of personality functioning, and maladaptive personality traits: mediation analyses in a clinical sample
Source: Front Psychiatry. 2026 Apr 30;17:1675044. doi: 10.3389/fpsyt.2026.1675044 (PMC13171586; doi:10.3389/fpsyt.2026.1675044)
Supplement: Supplementary file 2 [file Image1.pdf]

Supplement Figure 1. Model plot of Confirmatory Factor Analysis for Two Dimensional Machiavellianism Scale (TDMS).

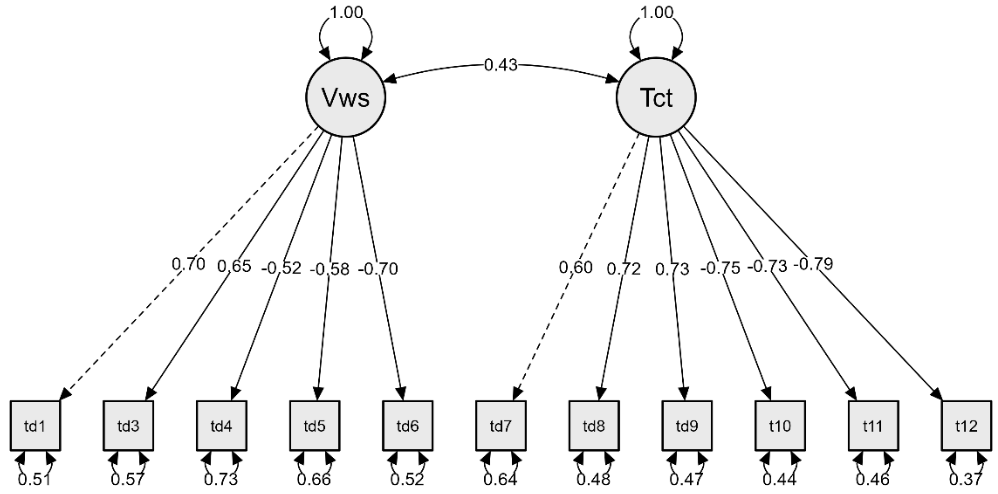

Note: Vws = TDMS Views; Tct = TDMS Tactics; td1 to td12 = TDMS items. All estimates are standardized.

Supplement Figure 2. Correlation heatmap depicting associations between measured variables.

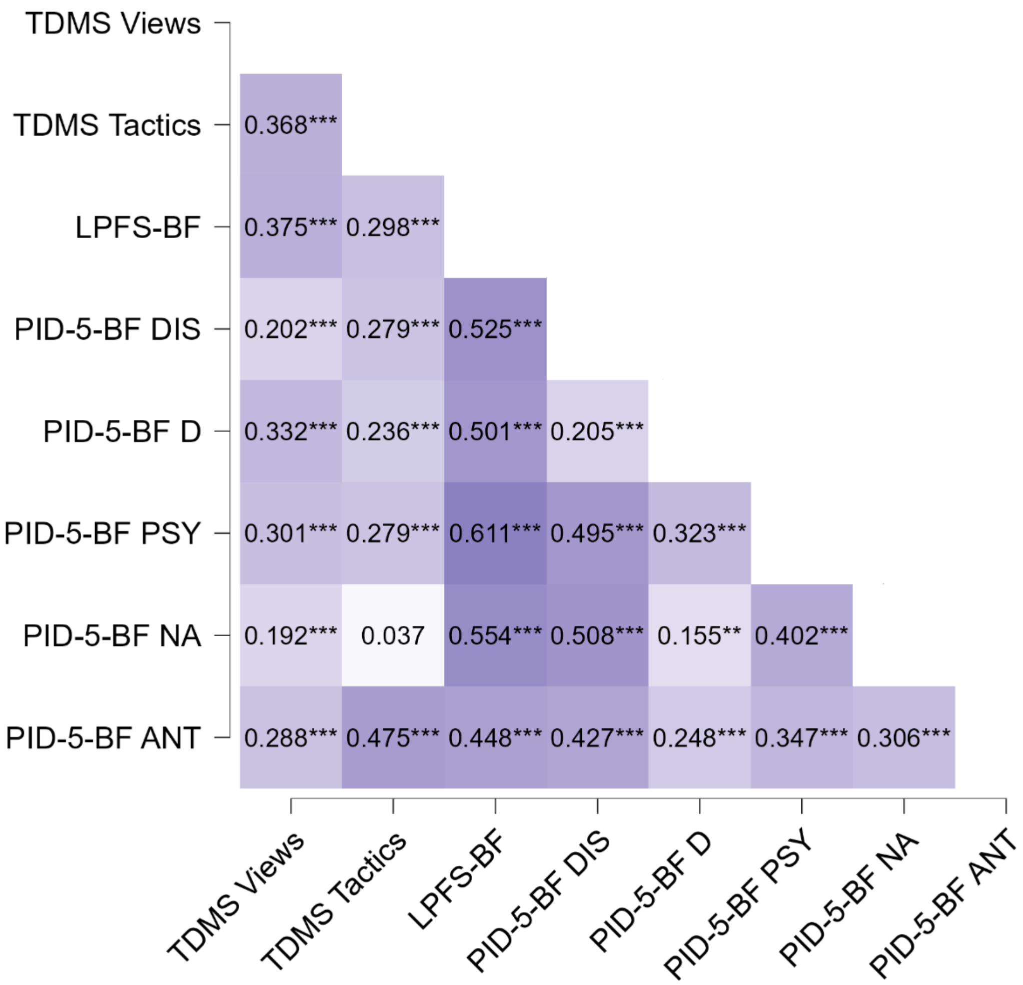

Note: Values represent Pearson correlation coefficients. Darker purple shades indicate stronger positive correlations. The asterisk indicates the level of significance. PID-5-BF = Personality Inventory for DSM-5 – Brief Form; DIS = Disinhibition; D = Detachment; PSY = Psychoticism; NA = Negative Affectivity; ANT = Antagonism; TDMS = Two-Dimensional Machiavellianism Scale; LPFS-BF = Level of Personality Functioning Scale – Brief Form 2.0.($p < .05$*, $p < .01$**, $p < .001$***).
